# Electrochemically grown porous platinum for electrocatalysis and optical applications

**DOI:** 10.1038/s42004-025-01476-4

**Published:** 2025-03-29

**Authors:** Sarmiza-Elena Stanca, Marco Diegel, Jan Dellith, Gabriel Zieger, Uwe Hübner, Andreas Ihring, Heidemarie Krüger

**Affiliations:** 1 Leibniz Institute of Photonic Technology, Jena, Germany; 2 Institute for Solid State Physics, Friedrich-Schiller University Jena, Jena, Germany

**Keywords:** Materials for energy and catalysis, Electrocatalysis, Electrochemistry, Materials for optics

## Abstract

Localized electrochemically grown porous platinum layers on 2D and 3D microstructured materials enable a wide range of applications from electrocatalysis to optoelectronics. These layers exhibit a thickness gradient and surface corner overloading due to electric charge accumulation at the sharp corners. On one hand, these effects can be applied to create ultra-large surface area catalysts or electrocatalysts. On the other hand, they can be mitigated by guiding the electric field at the nanoscale. Here, we show that porous platinum grown on rough conductive silicon synergistically catalyses the electroreduction of CO_2_ in a humid gaseous atmosphere, overcoming the disadvantage of CO_2_´s low water solubility. In addition, using template-directed growth of porous platinum, we tuned the optical response of an infrared (IR) metamaterial fabricated by micropatterning on Si/NiCr/Ti substrates and constructed a broad absorber on potential IR-functional microcomponents.

## Abstract (web summary)

Electrochemically grown porous platinum layers exhibit charge accumulation gradients. Here, the authors show that composite materials that rely on localized electrochemical growth of porous platinum can be applied as electrocatalysts for CO_2_ conversion, to tune the optical response of infrared metamaterials, and as broadband absorbers in optical sensors.

## Introduction

**Fig. 1:**
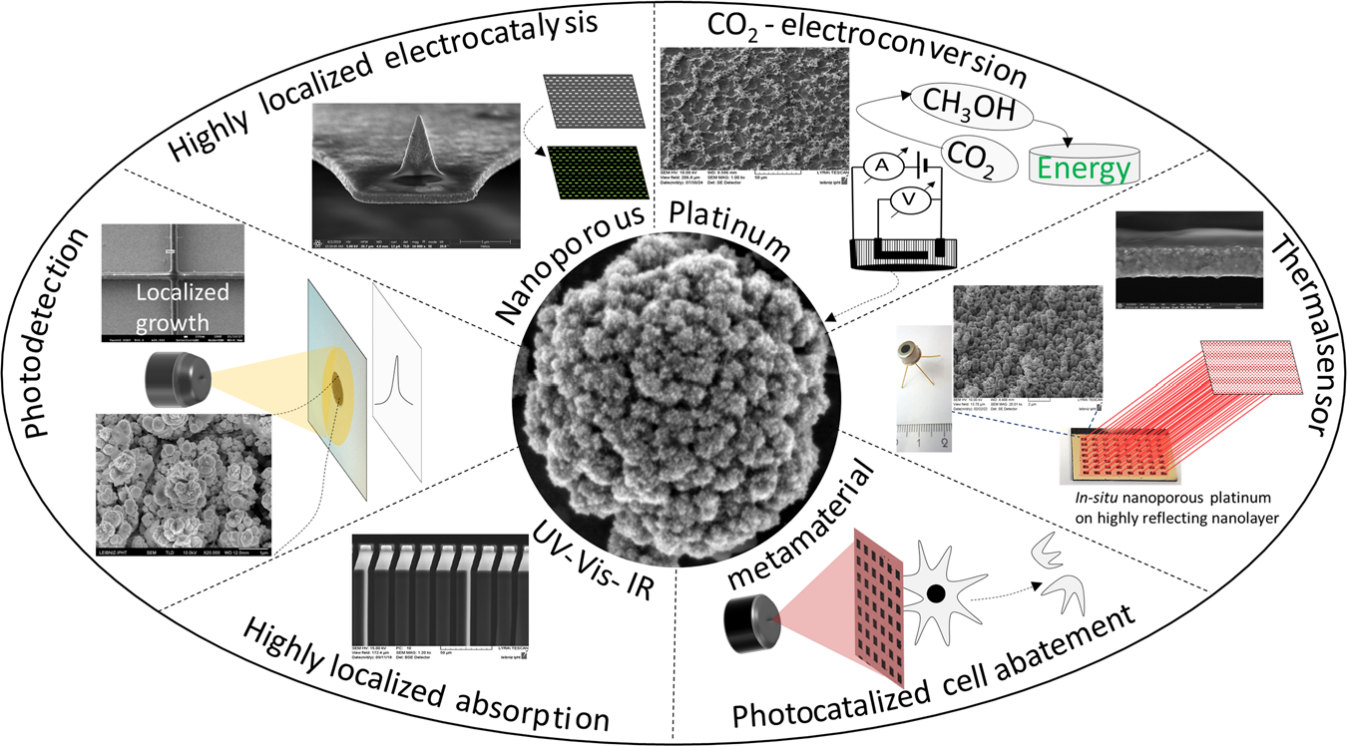
Selected use of localized electrochemically grown nanoporous platinum. Schematic representation.

The chemothermal stability in air of platinum empowers exclusive applications in metrology^1^, catalysis^2^ and optoelectronics^3^. The porous platinum thin layer of ultra-large surface area and good electrical conductivity enables its use as an efficient electrode material for electrocatalysis due to its excellent cycling and recovering performances. Moreover, by applying this porous platinum layer to economical materials such as silicon, silicon dioxide or carbon, one mitigates the issue of cost-effective precious metals while the heterogeneous catalytic activity and noble surface are safeguarded. For example, the heterogenous catalytic activity of the SiO_2_-supported Pt nanoparticles^4^, of Au‑M/SiO_2_ (M = Rh, Pd, Ir, Pt)^5^, of intermetallic platinum–cobalt electrocatalysts^6^, of platinum catalyst coupled with transition metal/metal oxide^7^ have been already demonstrated. Elaborated methods for covering porous metals other than platinum with layers of platinum are comprehensively described in literature^8,9^. In addition, the applications in optoelectronics and quantum devices require along to the material intrinsic characteristics, a nanoscale precision and accuracy, to prevent instabilities in electronic properties. Porous platinum layers-based optoelectronics have been progressively driven towards nanoscale, where infinite-small structural fluctuations can cause huge undesired deviations in properties. In this regard, controlling the relationship structure - properties is a critical technical request. The geometric properties of the porous platinum nanolayers play an important role in determining their optical properties. Therefore, structural tuning of porous nanolayers by judiciously designing the nanopore´s shape, size, periodicity and their edge geometry remain nowadays an intriguing challenge. Moreover, the insertion of the optical components into the microelectronics mostly requires a non-aqueous media for operation and a possibility of material nanostructuring. We developed the growth of porous platinum nanolayers in non-aqueous media using microelectrochemistry on highly localized areas, enabling a large pallet of applications (Fig. 1). Among them, in the following, we present the applications of electrochemically grown porous platinum´s that ranges from conversion of CO_2_ to tuning the metamaterial‘s optical properties and further to high absorption of infrared radiation when targeting certain microstructures with potential in thermal sensors. These applications are divided in the following three categories:

1. synergistic electrocatalyst to CO_2_ conversion by growing porous platinum on raw electrically conductive silicon;
2. adjuster of metamaterial´s optical properties by templated growth of porous platinum;
3. nanoscale localized absorber by electro-engineered porous platinum growth.

The structural and optical characteristics of the electrochemically produced porous platinum layers were examined by the following methods: Scanning Electron Microscopy (SEM), Focus Ion Beam (FIB) coupled with SEM, Atomic Force Microscopy (AFM), X-ray Diffraction (XRD), Energy dispersive X-ray analysis (EDX) and Fourier Transform Infrared Spectroscopy (FTIR) operated under the linearly polarized light.

## Results and Discussions

**Fig. 2:**
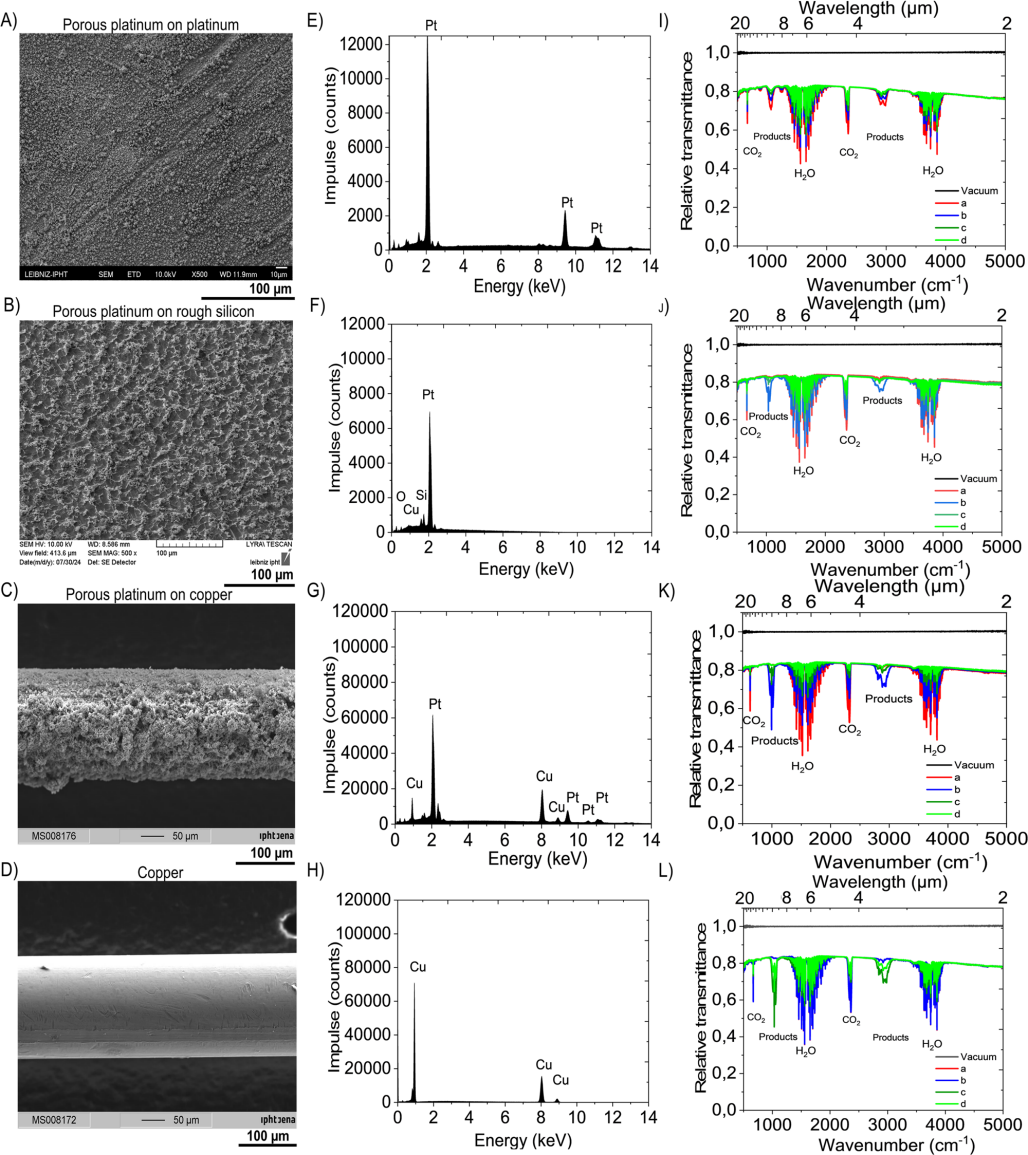
Electrocatalyst comparison. **A**–**C** SEM of the porous platinum on platinum (**A**), on rough conductive silicon (**B**) and on Cu wire (**C**); (**D**) SEM of Cu wire. **E**–**H** Corresponding EDX of the electrodes indicated in (**A**–**D**). The spectra were recorded at 15 keV or 20 keV, so that Pt-La,b lines are excited. The spectrum (**F**) was recorded at 10 kV to avoid Si large peak in the spectrum. The Pt-L lines are not excited and are therefore missing. **I**–**L** Corresponding raw FTIR transmittance spectra of the CO_2_ electrohemical conversion recorded on the electrodes indicated in (**A**–**D**) at 2 V potential difference between the anode and cathode at an interval of 10 s between the spectra a (red), b (blue), c (greem), d (light greem), respectively.

**Fig. 3:**
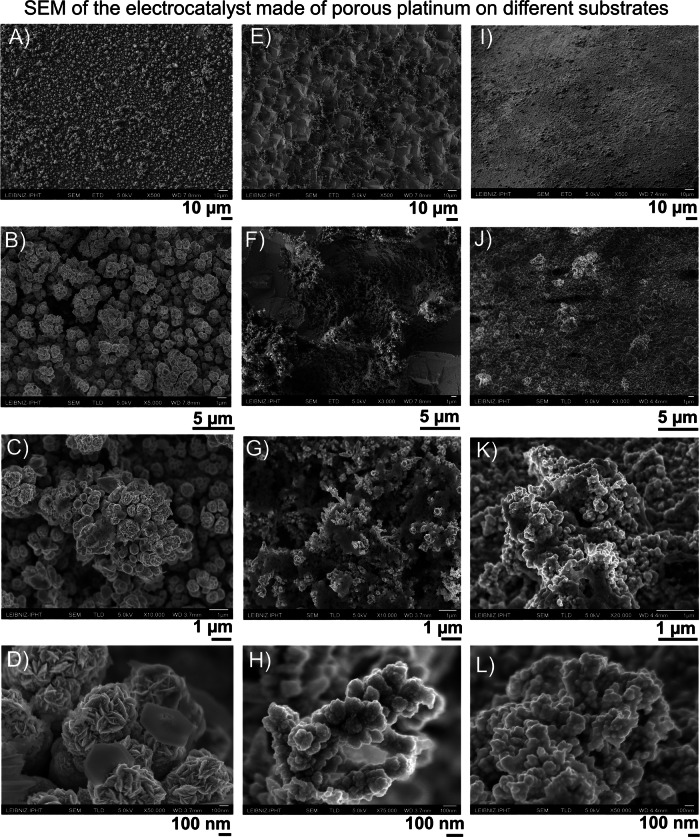
Electrocatalyst morphology. **A**–**L** SEM images at different magnifications of the electrocatalysts: porous platinum on platinum (**A**–**D**), porous platinum on conductive silicon (**E**–**H**), porous platinum on copper (**I**–**L**). The electrocatalyst nanostructure´s mean size extracted from the SEM images: porous platinum on platinum of 92 ± 10 nm, porous platinum on rough conductive silicon 77 ± 10 nm; porous platinum on copper 96 ± 11 nm.

**Fig. 4:**
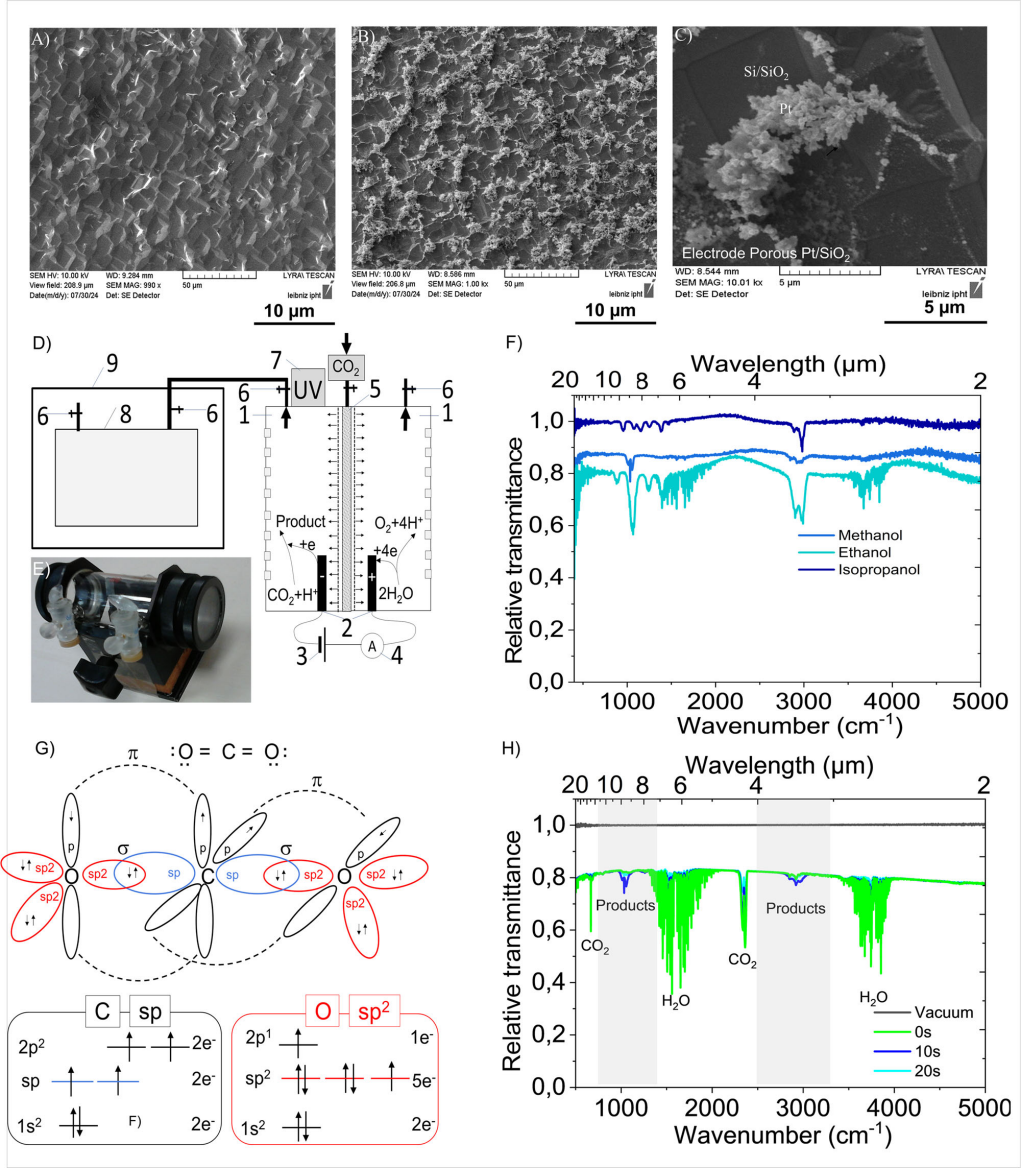
Porous platinum on rough silicon as electrocatalyst. **A**–**C** SEM images of the silicon void of porous platinum (**A**) and of porous platinum on silicon (**B**, **C**). **D** Spectroelectro-chemical setup for CO_2_ conversion. 1- chambers with humidity of 80–90%; 2- porous platinum grown on rough silicon electrodes; 3-direct current (DC) voltage source; 4-ampermeter; 5- oxygen impermeable membrane connected to CO_2_ inlet; 6- taps, 7-UV lamp for faciliting CO_2_ dissociation of 235 nm, 8–100 mL gas chamber equipped with KBr windows and taps, 9-spectrometer. **E** Photo of the FTIR-gas chamber equipped with KBr windows. **F** Transmittance spectra of the methanol, ethanol, isopropanol in the gaseous form. **G** The schematic representation of the carbon dioxide molecule, (ΔGf°=−394.39 kJ/mol) ^22^. **H** Transmittance spectra of the evacuated chamber (black line) and of the gas containing CO_2_ after applying ΔU = 2 V at 0 s (light blue line), 10 s (blue line) at 20 s (green line).

**Fig. 5:**
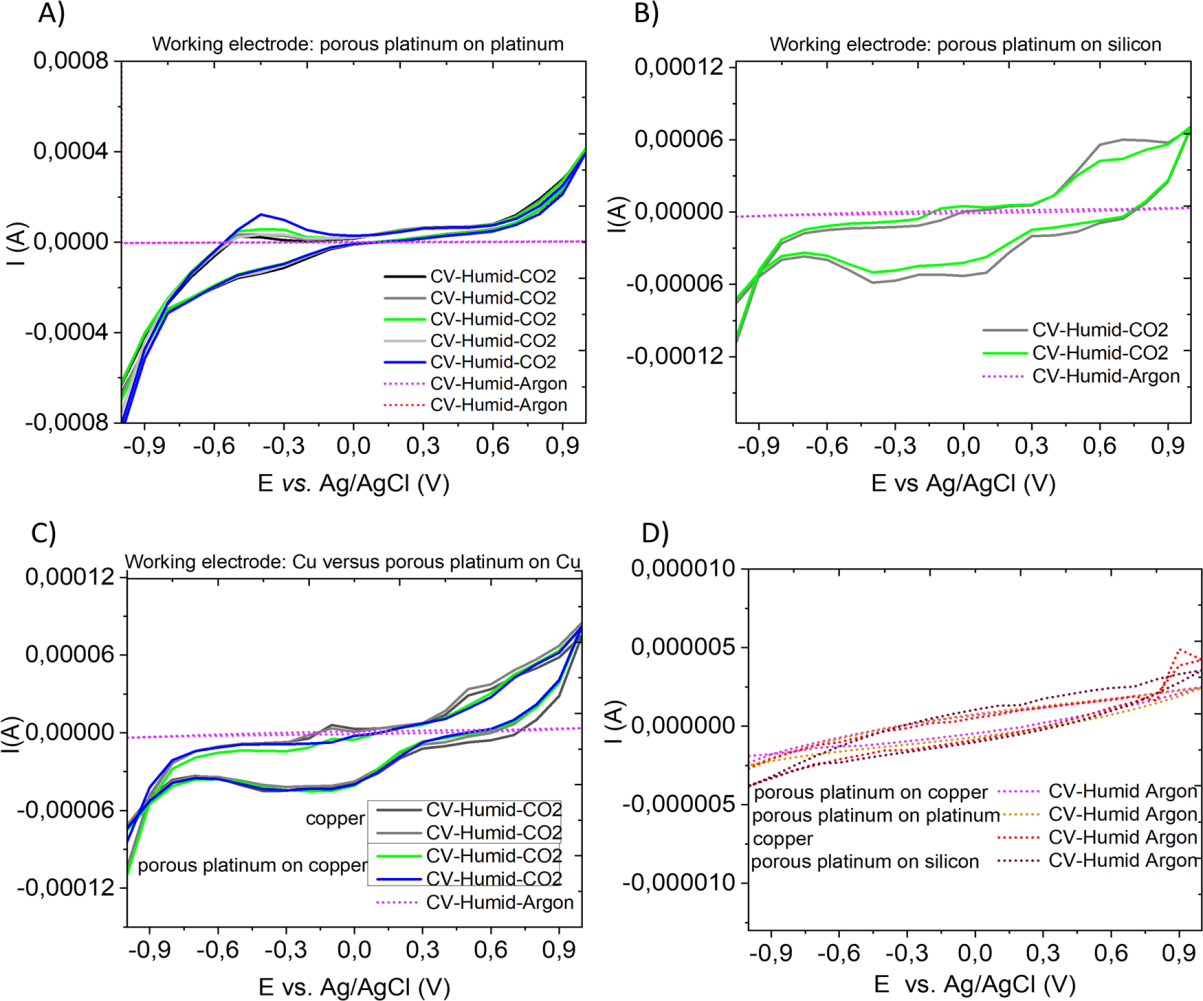
Electrochemical characterization of electrocatalysts. **A**–**D** Evolution of cyclic voltammetry (CV) recorded at 0.1 V/s, between +1 V and −1 V, in humid CO_2_ (**A**–**C**) compared with the cyclic voltammetry recorded on indicated electrodes in humid Ar (**D**). The amplified CV starts at +1 V. The diagrams (**A**–**C**) show the CO_2_ electroreduction activity of porous platinum on platinum (**A**), porous platinum on rough conductive silicon (**B**), copper (**C**), porous platinum on copper (**C**), respectively, as working electrode, Pt-counter electrode, Reference: Ag/AgCl.

**Fig. 6:**
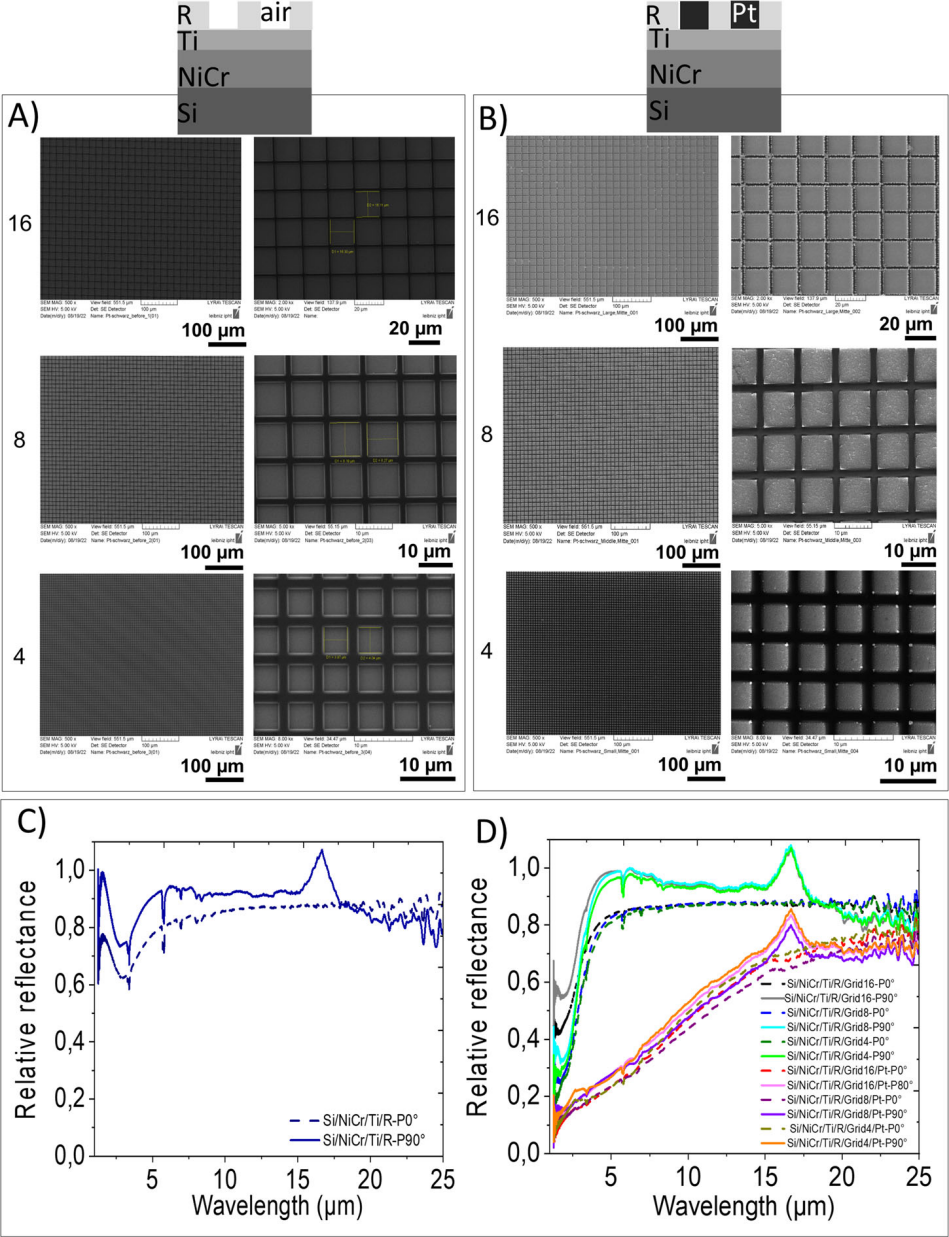
Analysis of the templated grown porous platinum in square-cavity-microarray. **A**, **B** Scanning electron microscopy of the samples before (**A**) and after (**B**) growing porous platinum on them; (**C**, **D**) FTIR relative reflectance spectra of the samples indicated in (**A**, **B**) recorded under 0° and 90° polarization. The spectra demonstrate the polarization dependent response of the resist (R) itself (**C**) and of the resist (R) polymer microstructured in square-cavity-microarray without/with porous Pt (**D**). Square cavity microarray are of 16 µm, 8 µm and 4 µm square ‘side, respectively.

**Fig. 7:**
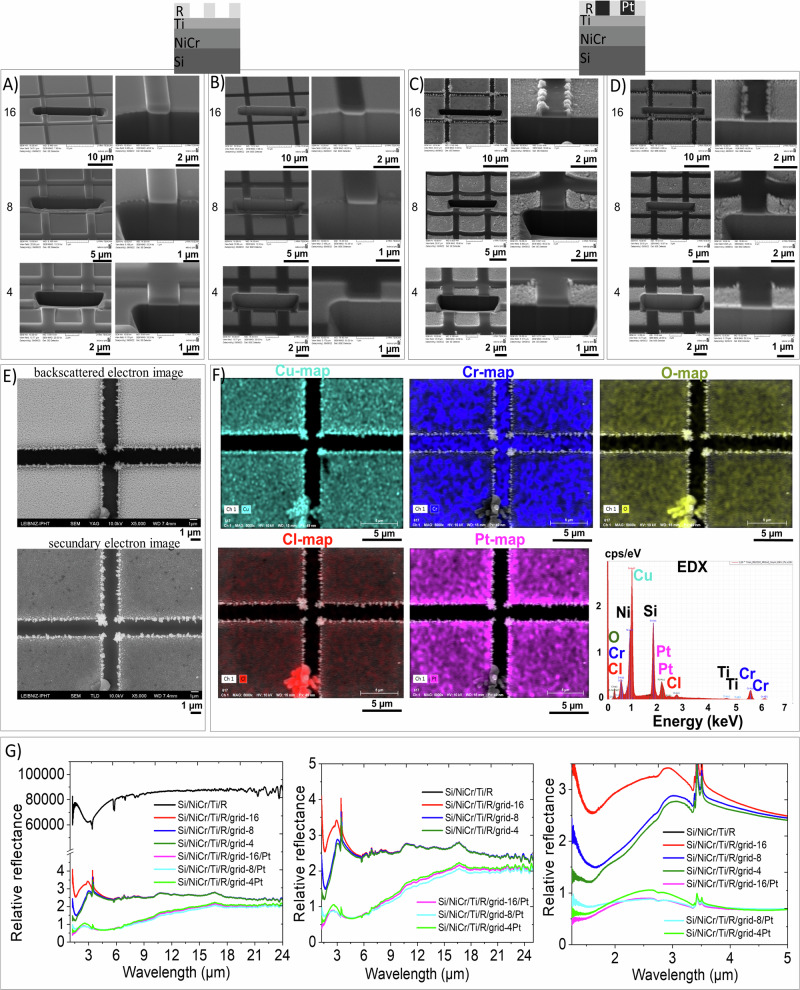
Analysis of the templated grown porous platinum in square-cavity-microarray. **A**–**D** Scanning electron microscopy images of the FIB cut samples before (**A**, **B**) and after (**C**, **D**) growing porous platinum on them. **E** Top view SEM images in both backscattered (up) and secondary electron (down) contrast modes of the area EDX mapped in (**F**). **F** EDX mapping analysis of the porous platinum templated grown in the square array and the corresponding EDX diagram. **G** FTIR relative reflectance spectra of the samples indicated in (**A**–**D**) recorded in the absence of polarizator. Square cavity microarray are of 16 µm, 8 µm and 4 µm square’side, respectively.

**Fig. 8:**
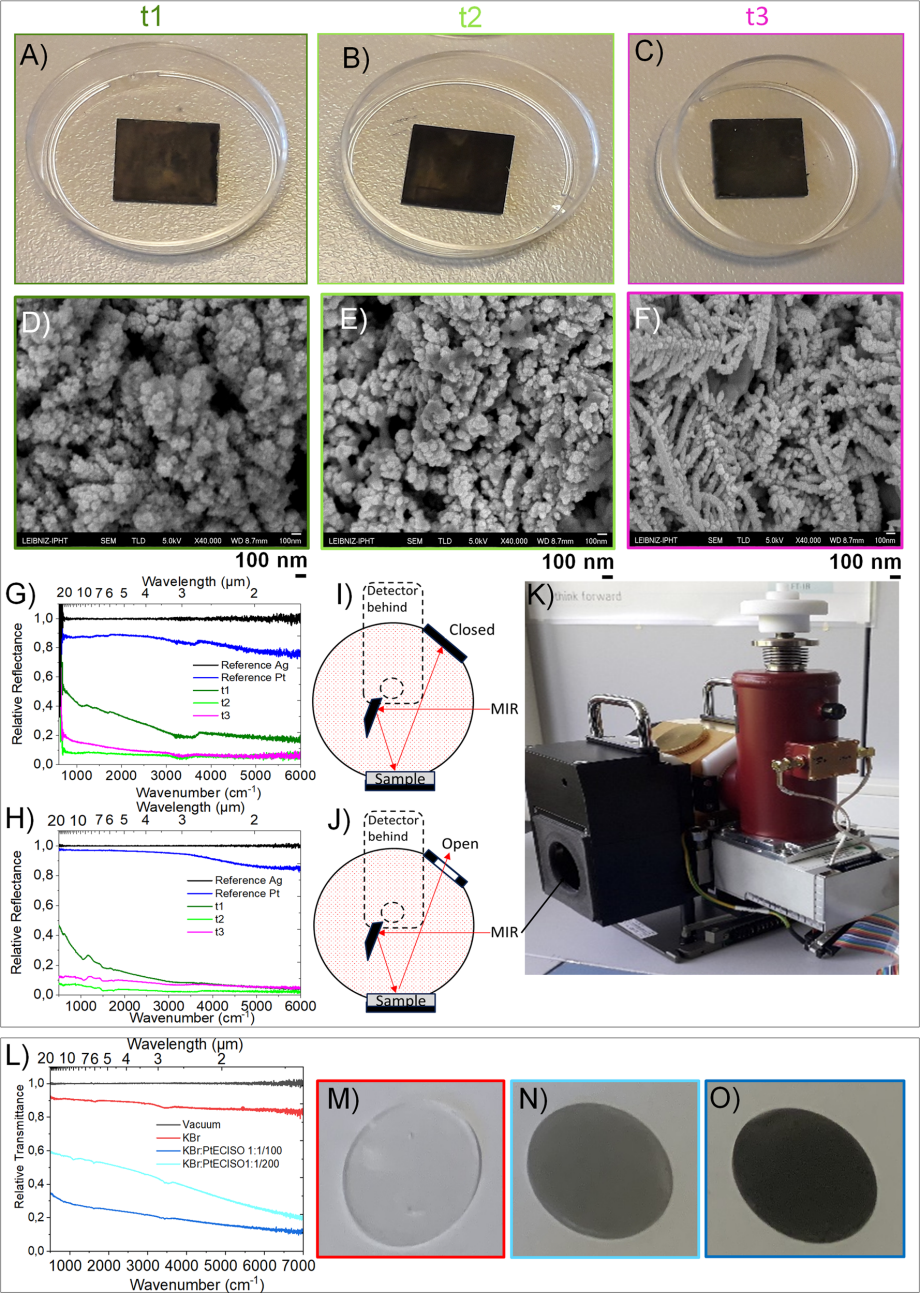
Characterization of the non-aqueous electrochemically grown porous platinum. **A**–**F** Porous platinum grown at three different electrolysis times (t1 = 60 s, t2 = 90 s, t3 = 160 s), photos (**A**–**C**) and SEM images (**D**–**F**). **G**, **H** The FTIR total reflectance (specular + diffuse reflectance) (**G**) and specular reflectance (**H**) spectra of the samples indicated in (**A**–**C**). **I**–**K** The sketch of total reflectance (**I**) and diffuse reflectance (**J**) and the photograph of the integrating sphere (**K**) used for this experiment; (**L**). Vacuum transmittance spectra of the pellet of composition indicated in diagram. **M**–**O** Photo on pellets of KBr (**M**) of KBr with porous platinum collected from the cathodes at the ratios of 1 g KBr:$\frac{1}{200}$ g porous platinum (**N**) and 1 g KBr:$\frac{1}{100}$ g porous platinum (**O**).

**Fig. 9:**
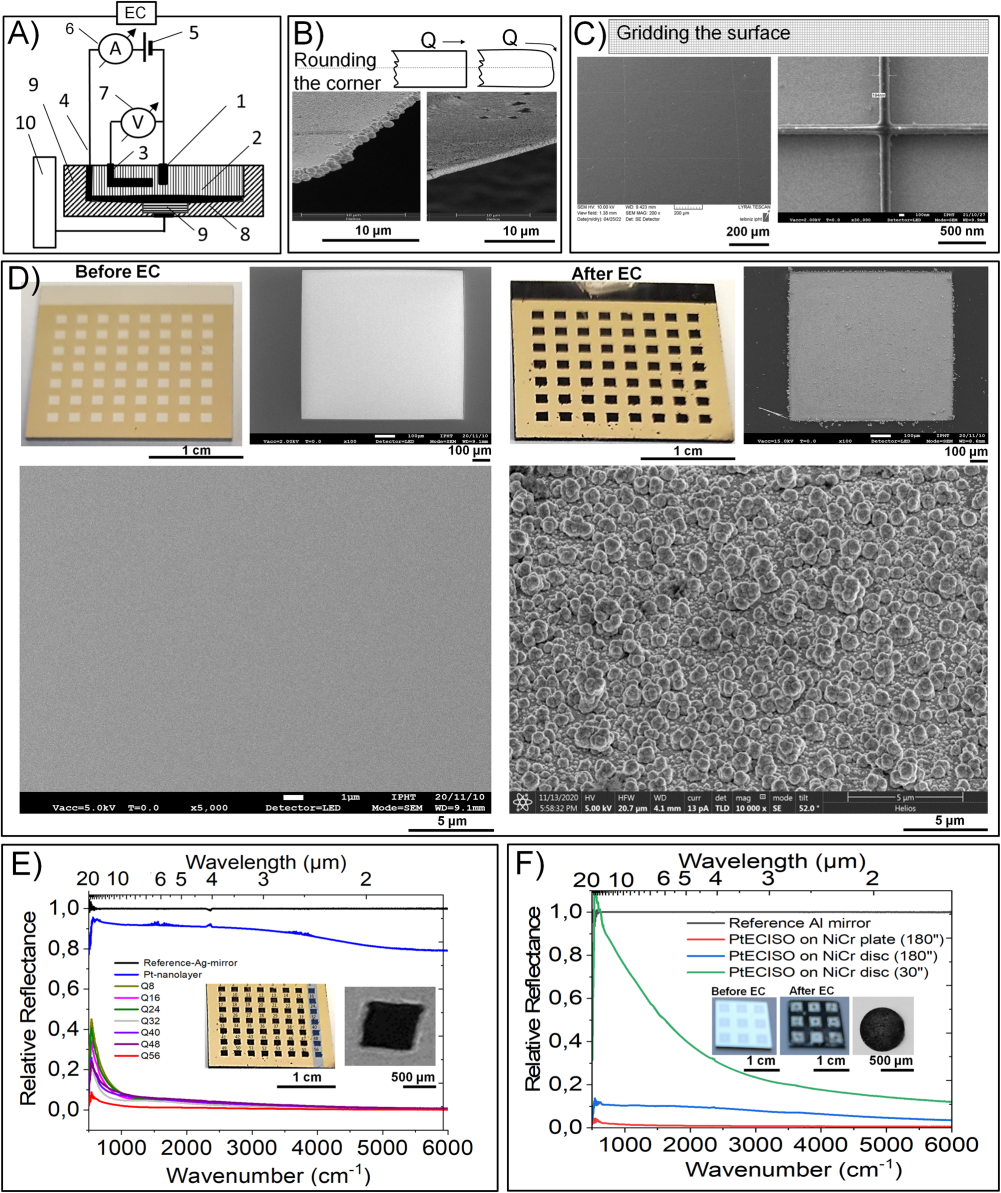
Nanoscale electro-engineered porous platinum growth. **A** Scheme of the electrochemical setup (EC):1-working electrode (cathode) consist of Si chips (preferably of 1.5 cm × 1.5 cm) modified with 110 nm Pt layer (optional masked), 2-Counter electrode (anode), 3-reference electrode (Platinum quasi-reference); 4-electrical conductors and connectors; 5- programmable source of current; 6-ampermeter; 7-voltmeter; 8-electrochemical cell recipient for the bath made of glass, 9-conector to thermostat for temperature control; 10-thermostat. **B** Rounding the margins to make the charge do not stop at the margins but to flow under the surface. SEM images of the AFM cantilevers with the rounded and not rounded margins. **C** Gridding the surface to disrupt the flow of the charge at the edge; SEM images of the gridded samples and a zoom at the grid cross. **D** Photographs and the SEM images of the gridded samples before and after porous platinum locally grown and used as broad-band absorber in the mid infrared wavelength region. The electrochemical growth is “EC” abbreviated. **E**, **F** FTIR reflectance spectra of the porous platinum electrochemically locally grown on square-shaped areas (**E**) and disc-shaped areas of 500 µm in diameter (**F**). The insets show the examined squares Q 8, Q 16, Q 24, Q 32, Q 40, Q 48, Q 56 in the photo of the square array as well as the photos of the disk areas on the chip.

For almost a decade, we have experimentally observed how the electrochemically grown porous platinum layers exhibit surface gradient and surface corner crowding as a result of the uneven distribution and accumulation of electrical charge at the edges and sharp corners of the surface. On the one hand, one can use this effect by creating a large-area catalyst/electrocatalyst (Figs. 2–4) which prove the electrocatalytic activity for CO_2_ reduction (Fig. 5), on the other hand, one can weaken this effect by finely grating the surface, rounding the edges, and guiding the electric field at the nanoscale to tune the optical properties of the localized platinum layers on 2D -, 3D-microstructured materials (Figs. 6–9). These approaches have enabled us to achieve broad and even divergent applications for porous platinum, which we summarize here and divide into three categories.

### Application of porous platinum grown on conductive rough silicon as electrocatalyst

Platinum is renowned for its catalytical performance; however, the high cost impedes it large use. By growing thin layers of porous platinum on economical materials such as silicon or silicon dioxide one increases the catalytic active area of noble metal guarantying the efficiency while mitigating the cost-effective. Reported here is the growth of localized porous platinum nanolayers on electroconductive shaped surfaces such as rough electrically conductive silicon to catalyze the simultaneous and separate production from water of O_2_ and H_2_ and further conversion of CO_2_ to beneficial chemicals.

### Porous platinum/Si/SiO_2_ synergistic electrocatalytic effect for CO_2_ conversion

The continued increase in atmospheric concentrations of CO_2,_ which in 2024 attained 37.4 billion metric tons^10^, motivates creatives to develop methods to mitigate its excess by conversion of CO_2_ in useful chemicals. The products of CO_2_ conversion as fuels or precursors for the production of more complex chemicals are now in the scientific focus. The low solubility in water of the CO_2_^11,12^ of 0.1449 grams CO_2_ per 100 ml H_2_O at 25 °C^12^ caused by the small polarization degree of the molecule, coupled with the high dissociation energy of CO bond in CO_2_ of 191 kcal.mol^−1^ = 799.144 kJ mol^−1^^12^ make the CO_2_ conversion at room temperature and atmospheric pressure a scientific issue nowadays. The stability of CO_2_ molecule is assured by the electronic distribution in the orbital architecture (Fig. 4G), a linear centrosymmetric structure of two similar C = O bonds. The central carbon participates in the CO_2_ molecule with two sp-hybridized orbitals and two p orbitals, where each orbital of them is occupied by one decoupled electron. Instead, oxygen participates in the CO_2_ molecule with one p orbital occupied by two coupled electrons and three sp^2^ orbitals, two of them occupied by four coupled electrons and the remaining sp^2^ orbital with an uncoupled electron (Fig. 4G). The orbitals occupied by the uncoupled electrons join together to form pi and sigma bonds, respectively, sigma bonds confer to the whole molecule a pronounced s-character, while the pi bound confer rigidity. In the typically sp² organic compounds, the dissociation energy of carbonyl bound C = O is 178 kcal mol^−1^ = 744.752 kJ mol^−1^^13^ while in CO_2_, the dissociation energy of CO bond is 799.144 kJ mol^−1^^14^. Despite of CO_2_ stability, the plants easily use the energy of the sunlight to photosynthesize glucose from water and CO_2_ absorbed from the air (Eq.1) at no high pressure and no high temperature.

1 $$n\,{{{\rm{CO}}}}_{2}+n\,{{{\rm{H}}}}_{2}{{\rm{O}}}\to {({{{\rm{CH}}}}_{2}{{\rm{O}}})}_{n}+n\,{{{\rm{O}}}}_{2}$$

This natural achievement is possible by using the light-driven electrochemical processes at the biological membranes^15^. The transfer of electrons in photosynthesis are assigned to be an electrochemical process, the oxidation of excited chlorophyll at an electron acceptor complex, which has the role of electrode. The membrane-bound electron transfer molecules play an active role in oxidizing an electron-donor molecule on one membrane side and reduce an electron-acceptor molecule at the reverse side. The structure of energy transducing membranes as a result of the organization of biological electron transfer molecules are key tools for facilitation of the photosynthetic reaction^15^. Mimicking the nature, experiments conducted under light irradiation showed the splitting of CO_2_^16–19^. The work carried out under the ultra violet (UV) light radiation demonstrated the CO_2_ photodissociation reactions (Eq 2. Eq 3):

2 $${{{\rm{CO}}}}_{2}\to {{\rm{CO}}}+[{{\rm{O}}}]$$

3 $${{\rm{CO}}}\to {{\rm{C}}}+[{{\rm{O}}}]$$

Equation 2 at 5.45 eV corresponding to 227.49 nm with the dissociation limit at 7.42 eV corresponding to 167.09 nm^16^ and Eq. 3 at 11.4 eV corresponding to 108.76 nm^16,17,19^. Furthermore, dissociation of carbon dioxide at room temperature upon the irradiation with a light at 213 nm and 266 nm has been reported^19^. An interesting approach infrared spectroscopically assisted of photocatalytic reduction of CO_2_ in aqueous electrolyte on SiO_2_/Fe_2_O_3_/Reduced Graphene Oxide (RGO) attained high conversion yields^18^. Furthermore, experiments with chemical^20^ or electrochemical^21^ routes conducted in aqueous electrolytes with catalytic materials from platinum to copper that facilitate adsorption, coupling, and hydrogenation via proton-coupled electron transfer processes demonstrated the reduction of CO_2_ into the valuable chemicals (i.e. methanol, ethanol). Because the standard Gibbs free energy formation of gaseous CO_2_ (Δ*G*_f_° = −394.39 kJ mol^−1^)^22^ shows a spontaneous redox reaction [C] + 2[O] = CO_2_, reducing CO_2_ requires an amount of energy equal to the standard Gibbs free energy. The standard Gibbs free energy is in a relationship with the potential of an electrochemical cell as follows in Eq. 4:

4 $$\Delta {{\rm{G}}}^{\circ} =-{{\rm{nFE}}}^{\circ} \;{{\rm{cell}}}$$

where n is the number of the transferred electrons, F-Faraday number 96,485 *J* V^−1^(mol e^−^)^−1^. E°cell-standard potential of the electrochemical cell (V).

The standard potential of CO_2_ reduction to CH_3_OH is very small 16 mV versus reversible hydrogen electrode (RHE) and 20 mV versus standard hydrogen electrode (SHE)^23^ (Eq. 5)) while for the reduction of CO_2_ to CO it is 103 mV versus SHE^24^ (6)

5 $${{{\rm{CO}}}}_{2}+{6{{\rm{H}}}}^{+}+{6{{\rm{e}}}}^{-}\to {{{\rm{CH}}}}_{3}{{\rm{OH}}}+{{{\rm{H}}}}_{2}{{\rm{O}}},\,{{{\rm{E}}}}_{0}=+0.020\,{{\rm{V}}}\; {{\rm{versus}}}\; {{\rm{SHE}}}$$

6 $$\begin{array}{cc}{{{\rm{CO}}}}_{2}+{2{{\rm{H}}}}^{+}+{2{{\rm{e}}}}^{-}\to {{\rm{CO}}}+{{{\rm{H}}}}_{2}{{\rm{O}}} & {{{\rm{E}}}}_{0}=-\,0.103\,{{\rm{V}}}\; {{\rm{versus}}}\; {{\rm{SHE}}}\end{array}$$

Despite of this small values, there are three major inconveniences connected to the CO_2_ electroreduction in aqueous electrolytes: 1) high overpotential which bring to a reduction process that overlaps the H_2_ electro-reduced evolution. 2) the low solubility of CO_2_ in water, 3) low selectivity in products. Therefore, there is a continued effort to find a highly active and efficient catalyst towards selective electroreduction of CO_2_^23–34^ since 1870, when the first electrocatalytic reduction of CO_2_ to formic acid was reported^33^. Products, such as monoxide carbon (CO)^26,35^, formic acid/formiate (HCOOH/HCOO^−^)^31,33^, formaldehyde (HCHO)^31^, methane (CH_4_)^29,30^, ethylene (C_2_H_4_)^28,30^, methanol (CH_3_OH)^23–36^ or ethanol (CH_3_CH_2_OH)^37^ were achieved. The reported work resumes at the electrocatalytic conversion of CO_2_ in aqueous electrolytes, nevertheless, recently it was demonstrated the advantage of inserting gaseous phase adjacent to the electrode^35^ or even to use vapor at 220 °C^38^ to increase the efficiency of electrocatalysis. Here we show a gaseous process at room temperature, UV light activated electro catalytical conversion of CO_2_ to CH_3_OH. In this direction, here, we compare the electro catalytical activity of the established electrocatalysts such as porous platinum on platinum, porous platinum on copper and copper void of platinum with the proposed one: porous platinum electrochemically grown on conductive rough silicon. Both morphological and composition analysis of the electrodes are presented in the Figs. 2,3. Figure 2A–D and Fig. 3A–O show the SEM images, while Fig. 2E–H shows the EDX of the used electrodes. By in situ FTIR analysis of the species produced at the electrocatalysts in a gas chamber equipped with KBr windows (Fig. 2I–L) there was observed a durable synergistic effect on the selective conversion of CO_2_ to the formation of methanol on porous platinum grown on the conductive silicon electrocatalysts (Fig. 2J, Fig. 4H).

The established electrodes such as copper, porous platinum and porous platinum/copper showed an expected electrocatalytic effect towards CO_2_ conversion (Fig. 2I–L, Fig. 5). Moreover, we demonstrate here that a high roughness electrode of porous platinum grown on conductive silicon synergistically works to convert CO_2_ to methanol. In addition, this method using the gaseous CO_2_ and micro-sized H_2_O droplets overcomes the drawback of low water solubility of CO_2_ and advantageously, can be in perspective connected with the silicon based green energy producer. We explain our finding by a three-microphase mechanism, the electrocatalytic reduction of CO_2_ takes place at the interface of solid catalyst with liquid water-droplet and gaseous CO_2_. This approach uses a 254 nm-UV line for stimulating the dissociation of CO_2_ coupled to an electrocatalytic process conducted into gas chamber under 80-90% humidity, for reducing CO_2_ to CH_3_OH. The electrochemical setup (Fig. 4D) connected to the spectrometer ´s gaseous chamber (Fig. 4E) equipped with KBr windows uses a composite cathode prepared by growing porous platinum on rough conductive silicon (Fig. 4A–C) (§Methods). The SEM image in Fig. 4B, C shows how abundantly grown porous platinum on rough silicon leaving blank certain areas of Si/SiO_2_, The EDX analysis has proven the presence of the following chemical elements: platinum, silicon, oxygen and copper (Fig. 2F). The mechanisms of CO_2_ electroreduction depend on several factors, among which the crucial roles are taken by the material type, morphology, temperature, electrolyte composition and their synergistic effects. We observed that SiO_2_ alone shows no catalytic effect on the conversion of CO_2_ to CH_3_OH at room temperature, however porous platinum grown on Si/SiO_2_ shows good efficiency to this reaction. Jiang et al. demonstrated that SiO_2_ support promotes a synergistic effect upon the bimetallic catalyst Pd/Cu at the CO_2_ conversion to methanol^36^. Moreover, to prove the role of SiO_2_ support in the global catalytic activity, Kusama et al. ^37^ reported the synergetic role of Rh-Li/SiO_2_ catalyst for CO_2_ conversion to ethanol/methanol via CO intermediate(s). Ton this basis, the following possible reactions can take place at the cathode:

7 $${{{\rm{CO}}}}_{2}+{2{{\rm{e}}}}^{-}\to {{\rm{CO}}}+[{{{\rm{O}}}}^{2-}];$$

8 $${{{\rm{CO}}}}_{2}+{{{\rm{H}}}}_{2}{{\rm{O}}}+{{\rm{Si}}}\to {{\rm{CO}}}+{{{\rm{H}}}}_{2}+{{\rm{Si}}}{{{\rm{O}}}}_{2},$$

9 $${{{\rm{CO}}}}_{2}+{{{\rm{H}}}}_{2}\to {{\rm{CO}}}+{{{\rm{H}}}}_{2}{{\rm{O}}},$$

10 $${{\rm{CO}}}+{{{\rm{H}}}}_{2}\to {{{\rm{CH}}}}_{3}{{\rm{OH}}},$$

11 $$2{{{\rm{CO}}}}_{2}+4{{{\rm{H}}}}_{2}{{\rm{O}}}\to 2{{\rm{C}}}{{{\rm{H}}}}_{3}{{\rm{OH}}}+{3{{\rm{O}}}}_{2};$$

while at the anode, the micro sized particles of water are oxidized to oxygen molecules:

12 $${{{\rm{H}}}}_{2}{{\rm{O}}}\to 1/2{{{\rm{O}}}}_{2}+{2{{\rm{H}}}}^{+}+{2{{\rm{e}}}}^{-}$$

To prove the electrocatalytic activity of the investigated electrodes, cyclic voltammetry of humid CO_2_ compared with the cyclic voltammetry in humid Ar were recorded and displayed in the Fig. 5. The diagrams indicate the evolution of cyclic voltammetry in humid CO_2_ (Fig. 5A–C) compared with the cyclic voltammetry recorded on indicated electrodes in humid Ar (Fig. 5D). The analysis of cyclic voltammetry shows that the CO_2_ reduction vs. Ag/AgCl at around −0.5 V on porous platinum/Pt, −0.4 V on porous platinum on conductive silicon or on both copper and porous platinum/copper (Fig. 4A–C). Practically, the electrolysis of CO_2_ using two electrode electrolysis in humid gaseous atmosphere requires a start difference of potential of 2 V. This experimental observation is supported by the electrolysis in the gaseous atmosphere at 160 °C achieved by Fischer et al. ^38^ at a difference of potential of 3 V–4 V on porous composite electrodes incorporating metallic particles of tin, bismuth, indium lead, respectively.

This work investigates the porous platinum grown on rough silicon ability to work as an efficient electrocatalyst. The electrochemical conversion of CO_2_ that occurs at the electrode/water/CO_2_ interface was demonstrated here using FTIR, as the difference between the gaseous chemicals can be detected by their infrared fingerprints. As reference spectra, the FTIR transmittance peaks of the gaseous methanol, ethanol and isopropanol in the gas chamber of the spectrometer, were respectively recorded, (Fig. 4F). With these references, the spectra of the conversion products were compared (Fig. 4H). FTIR transmission mode spectra shows the CO_2_ vibrational modes at 667 cm^−1^ (torsion vibration mode), 2341 cm^−1^ (C-O symmetric stretching modes), 2361 cm^−1^(C-O anti-symmetric stretching modes) before applying the electrical potential. After applying the potential difference of 2 V between the electrodes at a current density of 0.1 A/cm^2^ the CO_2_ conversion starts and is proven by the decreasing of corresponding intensity peaks while apparition of the additional peaks at 1033 cm^−1^, 1075 cm^−1^, 1145 cm^−1^_,_ 1233 cm^−1^, 1450 cm^−1^, 1470 cm^−1^, 2844 cm^−1^, 2970 cm^−1^, 3000 cm^−1^ 3080 cm^−1^. The vibrational modes correspond most probably to methanol^39,40^ because they match to the methanol peaks assigned as follows: v1 O-H stretch 3681 cm^−1^, v2 C-H anti-symmetric stretch (A’) 2999 cm^−1^, v3 C-H parallel symmetric stretch (A’) 2844 cm^−1^, v4 C-H in-plane anti symmetric bend 1477 cm^−1^, v5 C-H in-plane symmetric bend 1454 cm^−1^, v6 O-H bend (A’) 1340 cm^−1^, v7 CH3 rock (A’) 1074 cm^−1^, v8 C-O stretch (A’) 1033 cm^−1^, v9 C-H anti symmetric stretch (A”) 2970 cm^−1^, v10 C-H out of plane anti symmetric bend 1465 cm^−1^, v11 CH_3_ rock (A”) 1145 cm^−1^, v_12_ torsion 270 cm^−1^^41^. The vibrational mode O-H stretch at 3681 cm^−1^ of the methanol is not clear visible in our spectra because of the overlapping with the OH stretch bands of the water. These absorption bands are broadened by the intermolecular hydrogen bonding interactions between the hydroxyl groups causing a wider range of O-H stretching vibrations^42^. The steep distinctive peak at 1033 cm^−1^ in the products spectra, which is specific for methanol, indicates the presence of methanol in the gaseous products rather than ethanol or propanol. The faradaic efficiency (§Methods) shown as a ratio of peak intensity CO_2_ per CH_3_OH divided by the charge traverses the cell in time. The electrochemical approach connected to the FTIR demonstrates the efficiency of porous platinum grown on conductive silicon cathode (Fig. 2B) in wet gaseous media, for CO_2_ conversion (Fig. 2J). compared to the established electrocatalyst such as platinum (Fig. 2I)., copper/copper oxide (Fig. 2L). Table 1 summarizes a comparison of this work with the literature´s reported data on the CO_2_ electrocatalytic conversion to CH_3_OH, CO, HCOOH. in aqueous media^23–27,35^ and in vapor at 220 °C^36^.

**Table 1:** Faradaic efficiency of the CO_2_ conversion in CO, CH_4_, C_2_H_4_, CH_3_OH

Faradaic efficiency/Method	Electrode	Medium	Potential vs RHE (*E*_*RHE*_) or potential diff errrence (ΔU)	Product	Reference
81%/NMR	FeS_2_/NiS	Aqueous	−0,65 V	CH_3_OH	[23]
38%GC	Cu_2_O	aqueous (0.5 M KHCO_3_), pH 8.31	−0.36 ÷ 0.76 V*	CH_3_OH	[24]
−/GC	Cu_2_O	Aqueous	−0,50 V	CH_3_OH	[25]
80%/GC	Au-gas-electrodes**	aqueous, pH 2-4	2–6 V, 200 mA cm^−2^	CO	[34]
91%/GC	Au /18 °C***	aqueous 0.5 M KHCO_3_ pH 8.3	−1.1 V 3.7 mA cm^−2^,	CO	[26]
60%/EC	Cu	aqueous (KHCO_3_) 10 °C	−1.8 V 5 mA cm^−2^	CH_4_	[27]
20%/GC	Cu	aqueous (KHCO_3_) 40 °C	−1.33 ÷ −1.39 V 5 mA cm^−2^	C_2_H_4_	[30]
80%/ -balance	C/In, Sn, Pb, Cd, Tl nanoparticles	vapor at 220 °C	ΔU = 3 V 400 mA/cm^2^	HCOOH Crystals	[37]
68%/FTIR	Cu/CuO	gaseous/humidity 80–85% RT	ΔU = 2 V & UV 100 mA/cm^2^	CH_3_OH	this work
65/%/FTIR	Porous Pt/Cu	gaseous/humidity 80–85% RT	ΔU = 2 V & UV 100 mA/cm^2^	CH_3_OH	this work
70/%FTIR	Porous Pt/Pt	gaseous/humidity 80–85% RT	ΔU = 2 V& UV 100 mA/cm^2^	CO CH_3_OH	this work
72%/FTIR	Porous Pt/rough e^-^ conductive Si	gaseous/humidity 80–85% RT	ΔU = 2 V&UV 100 mA/cm^2^	CH_3_OH	this work

The reversible hydrogen electrode (RHE).

E_RHE_ = E_Ag/AgCl_ + E°_Ag/AgCl_.

E_RHE_ is the potential versus RHE converted from the potential recorded *vs*. Ag/AgCl reference or from calomel electrode (SCE). E _Ag/AgCl_ is the external potential measured against the Ag/AgCl reference electrode. We consider the calculation uses the standard potential of Ag/AgCl, E°_Ag/AgCl_ = +0.1976 V. * potential vs.SCE [mercury (Hg)/calomel (Hg_2_Cl_2_) electrode]: E (Hg/Hg_2_Cl_2_) = −1.1 V ÷ −1.5 V.

E_RHE_ = E (Hg/Hg_2_Cl_2_)+E° (Hg/Hg_2_Cl_2_)+0.0591 pH = −1.100 + 0.242 + 0.0591*8.31 = −0,366 V.

E_RHE_ = E (Hg/Hg_2_Cl_2_)+E° (Hg/Hg_2_Cl_2_)+0.0591 pH = −1.500 + 0.242 + 0.0591*8.31 = −0,767 V.

E_RHE_ is the potential versus RHE converted from the potential recorded *vs*. calomel electrode (SCE). E (Hg/Hg_2_Cl_2_) is the external potential measured against the calomel reference electrode Hg/Hg_2_Cl_2_. We consider in our calculation the standard potential of Hg/Hg_2_Cl_2_, E° (Hg/Hg_2_Cl_2_) = 0.242 V at 25 °C. **CO_2_ converted to CO on Au gas-diffusion electrodes on three phase interfaces (gas-liquid-solid). ***CO_2_ converted to CO on Au electrode at 18 °C.

### Application of porous platinum to tune optical properties of IR metamaterial

Transformation of materials from visible to invisible state and vice versa is a perpetual desire of humanity. At the beginning of the 20^th^, the Maxwell equations^43^ gave optimism to this desire and predicted that the light can be differently than usual propagated into a certain material, called metamaterial, for the frequencies where electric permittivity (ε) and magnetic permeability (µ) of the material are negative^44,45^. Since that moment on, several investigations made steps of progress in finding particular conditions of anisotropy to fulfil the Maxwell equation of negative permittivity and permeability^44–63^. The desired surface plasmon polariton resonances depend on the intrinsic properties of the material and the geometry of the nanostructures. Such materials were created by structuring arrays of metallic shapes such as square^61^, prism^54^, wires^56^, coil piles^47,55^ and rod pair^58,62^ into the dielectric medium. The first experimental demonstrations of such behaviour in the optical visible range were accomplished, nearly at the same time, for pairs of metal rods into the dielectric material^53,58,62^ and of pairs of dielectric cavities in metal^51^. Later on, negative epsilon material was achieved in visible range (514 nm, 501 nm, 496 nm, 488 nm, 476 nm) with the use of a prism shaped ultrathin Au-Si_3_N_4_-Ag waveguide sustaining a surface plasmon polariton mode with antiparallel group and phase velocities^54^. Yao constructed Ag vertical organised nanowires (60-nm wire diameter and 110 nm center-to-center distance) in dielectric medium of Al_2_O_3_, which exhibit negative epsilon at the wavelengths of 660 nm and 780 nm^58^. For microwave frequencies of 8 to 12 GHz Shelby constructed unconventional material that consists of discrete scattering elements, that may be approximated as an effective medium for wavelengths that are larger than the unit cell size dimension of 5 mm^47^. Grady has demonstrated ultrathin terahertz polarization converters that are capable of rotating a linear polarization state into its orthogonal one^58^. On the basis of these results, structures of negative epsilon at 1.4 THz corresponding to the wavelength of 214 µm have been created. The gold cut-wire (length 82 µm and width 10 µm) array and the gold ground plane are 200 nm thick, and they are separated by a polyimide dielectric spacer with thickness ts=33 µm at the periodicity of 68 µm. The incidence angle of 25°, and the incident electric field E_0_ linearly polarized in the x direction (s-polarized), with an angle a = 45° with respect to the cut-wire orientation assure the atypical terahertz refraction. Under an incidence of a femtosecond laser pulses at 1.5 µm wavelength Dolling obtained a negative epsilon material using a squared structured solid of thicknesses of 85 nm, the square lattice with lattice constant a = 600 nm^51^^.^ Yu reported negative refraction of the collimated quantum cascade laser (λ_0_ = 8 µm) using plasmonic interfaces, consisting of an array of “V”-shaped-antennas that convey abrupt phase shifts in the optical path^57^. This breaks the constraint of standard optical components, which rely on gradual phase accumulation along the optical path to change the wave front of propagating light that the phase velocity can be negative and that group velocity can also be negative simultaneously. For attaining simultaneously negative ε and µ two separate resonances for these parameters in the refracting materials are required. Pendry^55^ circumvented this condition by introducing the idea of “resonant dipoles into a chiral medium” that splits the resonant transverse bands and results in a range of frequencies below w0 in which negative refraction can be seen for one of the polarizations. All the properties predicted for an isotropic nonchiral medium with ε < 0, and µ < 0 will be reproduced in this medium but only for one of the polarizations. This previews a huge resolution progress ability to focus the near fields and hence reproduce an image with resolution unlimited by wavelength. Inspired from the large amount of reports on this topic in visible range and motivated by the small amount of reports in IR range, we planned to show the capability of templated electrochemically grown porous platinum to tune optical properties of IR-metamaterial. For preparing the IR-metamaterial, we used the procedure described in §Methods. The cathodes which consist of Si/NiCr/Ti/ microstructured resist were prepared using sputtering and lithography as described in the § Methods. The thicknesses of the substrates: Ti (Supplementary Figs. 1, 2) and NiCr (Supplementary Figs. 3, 4) were evaluated using XRR. The templated electrochemically grown nanoporous platinum layer was analysed using scanning electron microscopy (SEM) and Fourier Transform Infrared Spectroscopy (FTIR) operated under linearly polarized light. In the control experiments, we observed that the resist ARP6200.13, which consists of a mixture of two polymers: polymethyl-alpha-chloracrylate and poly (alpha-methylstyrene) presents itself a polarization dependent behaviour in the wavelength regions 15–17 µm (Fig. 6). By structuring this resist polymer, a polarization-dependent optical response appeared in a wavelength region of 2.5–3.5 µm (Figs. 6,7). By filling the cavities with porous platinum, the spectral shape is significantly changed while the polarization-dependent optical response is preserved.

### Application of porous platinum as defined localized optical IR absorber

Specific sensing and filtering optical signals make use of various absorber configurations. The applications of optical absorbers in nanotechnologies where the dimension is an issue, demand modifications to the conventional absorber configurations. Nanoporous platinum layers has driven interest in nanotechnological optics due to its unique chemo-mechanical stability and broadband absorption. We opted for the noble material because it confers long term chemical stability and robustness. Moreover, due to a very small concentration of PtCl_4_ salt the method is also cost effective. Indeed, there are useful scientific reports of ultrathin layer absorbers^64–68^ prepared by doping an ultrathin silicon film^64^ or by combining noble nanostructures with established oxides such as indium-tin-oxide^67^, or by exploiting the in-plane birefringence of α-MoO_3_^68^ or even from polymers such as poly methylmethacrylate (PMMA)^65,66^. All these approaches attained subwavelength thickness (~nm) into a bi- or multilayer architectures. Our approach of one-step electrochemical fabrication of the thin films requires a small amount of noble salt, which also enables a relatively accessible, low-cost process. Therefore, porous platinum remains attractive compared to other materials in terms of both manufacturing costs and optical performance and stability. Organic absorbers are interesting from an optical point of view, but they are unstable at high temperatures, whereas platinum has good thermal and chemical stability in air. Figure 8A–C shows the photographs of porous platinum films grown on highly reflective platinum nanolayer (Supplementary Figs. 5, 6) at three different electrolysis times. The XRD analysis of the porous platinum confirm the crystalline nature of it (Supplementary Fig. 7). Figure 8D–F shows the corresponding SEM images, and Fig. 8G, H shows their low reflectivity. The integrating sphere experiments (Fig. 8I-K) proved that the low reflectance is rather due to absorption than to diffuse reflectance. Furthermore, the vacuum-transmittance spectra (Fig. 8L) of the porous platinum entrapped into the KBr pellets (photos in Fig. 8M–O) show accentuated absorption of the IR light. This method can be extended to other metals. Porous noble metals other than platinum can also be prepared electrochemically from a water-free solution as broadband absorber layers. Electrochemistry is possible in anhydrous solvent, if the solvent is electrically conductive after the salt addition (e.g. 0.05–1.0 g/g % metallic salts of Pt, Au, Pd, Ru, Rh, Os, Ir, Ag, Hg and Ti in solvent). The electric conductivity of the anhydrous solvent e.g. alcohol (ethanol, isopropanol), amines (ethylenediamines), aromatic amines (aniline), carboxylic acids (formic acid, propionic acid), esters (butyl acetate, ethyl acetate), sulfoxides (dimethyl sulfoxide), ketones (acetone), increases by the addition of certain metallic salts. In this way, we demonstrated that the metal salt increases the electrical conductivity of the low conductivity solvent such as isopropanol^69–71^ and can meet the condition of electrochemical production of noble metal black. Noble metal black^72–74^ represents an appreciated optical absorber material with the potential for use as a thin layer in optical sensors. In most cases, the insertion of the optical absorber in the construction of microelectronics requires a non-aqueous media for operation and a prospective that the material can be brought into a thin-layer. Therefore, an in-situ electro-deposition of optical absorbers on the constitutive microcomponents of the optical sensor (i.e. on metallic multi-junction), in non-aqueous media is of practical interest. Optimizing existing thermoelectric sensors to achieve desired higher absorption of infrared radiation and stability is an ongoing process. Currently, optical absorbers in the wavelength range from 0.4 µm to 20 µm are based on silver black^75,76^. However, silver black exhibits undesirable chemical reactivity toward air components^75,76^, affecting its long-term stability and performance, important properties for microelectronics technology. Our solution is to utilize porous noble metals^69–71^, to achieve the following advantages: 1) Synthesis in non-aqueous media; 2) high localization using in situ electrodeposition; 3) wide absorption and low reflection from ultraviolet to infrared range; 4) chemical stability in air; 5) mechanical stability and robustness; 6) grown on 2D and 3D conductive materials such as: NiCr8020, NiCr5050, Ti/Ni, Indium-tin oxide, tin-copper alloy, aluminum, copper, gold, platinum, silver^70^; 7) controlled thickness from 100 to 600 nm. The Fig. 9 shows the electrochemical controlled growth of porous platinum using the setup from panel (A) on engineered surfaces such as rounded corner cantilever (B) or gridded surfaces (C) as well as the highly localized electrochemically growth of porous platinum layers on inside of the square-cavity-array (D) made in resist on highly reflecting platinum nanolayer. The square array made in resist, a mixture of two polymers: polymethyl-alpha-chloracrylate and poly (alpha-methylstyrene), which can be lithographically microstructured (https://www.allresist.com/wpcontent/uploads/sites/2/2016/12/allresist_produktinfos_ar-p6200_englisch.pdf; http://www.lithoguru.com/scientist/lithobasics.html) (§Method). The high absorbance optical achievement of the porous platinum localized growth was investigated by FTIR. On one chip of 1.5 cm x 1.5 cm there are 56 square-cavities. The square cavity of 800 µm x 800 µm is electrochemically filled with porous platinum. The squares on one chip are counted from the left-up side (square1 named Q1) to the right down side (square 56 named Q 56). The Fig. 9E shows the FTIR spectra of the samples Q 8, Q 16, Q 24, Q 32, Q 40, Q 48, which achieved reproducible reflectance drops of 85% in the reciprocal wavenumber range 500-3500 cm^−1^ and 85–75% in the reciprocal wavenumber range 3500–6000 cm^−1^; Sample Q 56 showed higher reflectance drops 87-90% in the reciprocal wavenumber range 500–3500 cm^−1^. Moreover, Fig. 9F shows the FTIR reflectance spectra of the porous platinum electrochemically locally grown on disc-shaped areas on the chip. These results pave the way towards to the application of porous platinum as wideband absorbers on micro-structured/nano-structured surfaces, on miniaturized sensor components, respectively. If the resist is removed the meta-activity is changed while keeping the photoresist filled with porous platinum, the polarization dependence optical response can be utilized in the fabrication of the selective meta active optical sensors.

## Conclusions

We presented here three application categories of the localized electrochemically grown porous platinum in non-aqueous media. 1) We made use of the electrochemical corners overloading effect to grow porous platinum on conductive rough silicon. This porous platinum-Si/SiO_2_ composite material proved to be a synergistic, efficient, selective and economical electrocatalyst for CO_2_ conversion as well as for acidified water conversion in H_2_ and O_2_. The approach of CO_2_ conversion from humid air has potential for being efficiently coupled to the silicon based solar cell for green energy producing in parallel with the mitigation of CO_2_ pollution. 2) The optical response of the IR-metamaterial prepared by shaping the resist on Si/NiCr/Ti substrates into a square-cavity-array was transformed by filling electrochemically the cavity-array with porous platinum. 3) We used the templated growth of the localized porous platinum layers on micro-structured areas to locally tune their optical properties.

## Methods

### Sputtering method

#### Platinum nanolayer on Si substrates

Pt nanolayers of 100 nm thicknesses were deposited on silicon wafer substrates by the mean of sputtering method (BesTech Jena Sputtering System II, Jena, Germany) in the clean room at the Leibniz Institute of Photonic Technology Jena. Si wafer has been cleaned by Ar ions before Pt deposition.

#### NiCr/Ti nanolayers on Si substrate

Sputtering instrument (BesTech Jena Sputtering System II, Jena, Germany) in the clean room at the Leibniz Institute of Photonic Technology Jena was used to grow NiCr 50:50 nanolayers of 102 nm (Supplementary Figs. 3, 4) thickness and Ti nanolayers of 8 nm thickness (Supplementary Figs. 1, 2) on silicon wafer substrates. Prior deposition, Si wafer has been cleaned by Ar ions.

The AFM probes were previously covered with a layer of NiCr:5050 by sputtering before electrochemical deposition of porous platinum.

#### Conductive silicon substrate

Rough silicon substrates measuring 1.5 cm x 1.5 cm each with a resistance of 290 ohms were prepared at Leibniz-Institute of Photonic Technology Jena.

### Lithography

Si substrate/NiCr/Ti multilayers were covered by spin coating with resist (positive E-Beam resists AR-P 6200.13) (https://www.allresist.com/wp-content/uploads/sites/2/2016/12/allresist_produktinfos_ar-p6200_englisch.pdf) while reflecting layers of platinum on Si was similarly covered with AZ1505 photoresist and afterwards lithographically micro-structured (http://www.lithoguru.com/scientist/lithobasics.html) in a clean room at the Leibniz Institute of Photonic Technology, Jena, Germany. The wafer previously covered with resist was heated and then exposed to an E-beam in the case of E-beam resist and to a UV light in the case of photoresist.

### Localized electrochemical growth of porous platinum

99% PtCl_4_ (MW 336.89 g/mol; Article number CC22008) was supplied by Carbolution Chemicals GmbH, St. Ingbert, Germany. Pb(CH_3_COO)_2_ (Article number 1073750250) was supplied by Merck, Darmstadt, Germany. Localized electrochemical growth of porous platinum thin layer was achieved using a water-free electrochemical reduction of platinum ions on cathodes. To synthesize localized porous platinum layers, 0.05% PtCl_4_ in isopropanol was used as main component of the electrolytic bath. 0.001% Pb(CH_3_COO)_2_ or 0.001%Cu(CH_3_COO)_2_ in isopropanol was used as additive^69–71^. Platinum plate serves as the anode. The cathodes were: electrically conductive silicon, platinum, copper or micro-structured SiNiCrTi-resist in form of 4 µm x 4 µm square array, 8 µm x 8 µm square array, 16 µm x 16 µm square array substrates and also Si/Pt/resist 800 µm x 800 µm square array. The resist was not removed after the electrochemical deposition of porous platinum. Porous Pt does not grow on the resist (Fig. 7C, D).

### FTIR spectroscopy

The FTIR spectra in transmission mode or in reflection mode were recorded in a FTIR-Spectrometer (Vertex 80vBruker Instrument) at a resolution of 2 cm^−1^ as an average of 32 spectra in the spectral range 7500–500 cm^−1^. The samples were irradiated with a MIR silicon carbide light source of 6 mm aperture through a KBr-beam splitter. The spectra were collected with a standard FTIR detector with Mercury Cadmium Telluride (MCT) diode (D*: > 2 × 10^10^ cm Hz^1/2^W^−1^) liquid nitrogen cooled at 20kHz- mirror frequency or with Deuterated L-alanine Triglycine Sulphate (DLaTGS) room temperature operated (D*: > 2.7 × 10^8^ cm Hz^1/2^W^−1^). at 10 kHz- mirror frequency, 3 mm sample aperture. The measurements were conducted in vacuum at 4 hPa where not otherwise specified. For the total reflection and diffuse reflection measurements, the spectrometer was equipped with a gold coated integrating sphere of 150 mm diameter (type A562, Bruker Instrument) mounted on a QuickLock (QL) base plate. The IR beam impinges on the sample with an incidence angle of 13°. The diffusely and the specularly reflected light is detected by an opening sleeve of 7 mm with an MCT detector. For the polarization dependence investigation, the spectrometer was equipped with a reflection grazing unit of 80° incidence (type A518/Q, Bruker Instruments) mounted on a QuickLock (QL) base plate and a MIR polarizer (type I7590 ECL, Bruker Instruments). The spectro-electrochemistry investigations in transmission mode used a 100 mL gas chamber equipped with two taps and KBr windows mounted on a QuickLock (QL) base plate. For recording localized FTIR spectra on microstructures of square-array or disc-array were collected from an area of 300 µmx300 µm using a Hyperion Microscope with 15x objective Ser. 918 17425 (NA = 0.4) connected to Bruker Vertex80v Spectrometer operated in reflection mode, detector MCT-N2 spectral resolution 2 cm^−1^, mirror frequency 20 kHz, 32 spectra/sample. Data analysis were done using OPUS software and displayed with the OriginLab software.

### Scanning Electron Microscopy (SEM)

A JSM-6700F field emission microscope (JEOL, Tokyo, Japan) and FEI Helios NanoLab G3 UC (ThermoFisher, Netherlands) were used for most SEM measurements. Some of the micrographs were taken with a table tilt of 45°. The system is equipped with secondary electron detectors (SEI) of the InLens and Everhart-Thornley types, as well as detectors based on semiconductors and yttrium aluminum garnet (abbreviated as YAG type) for backscattered electrons.

### Focused ion beam (FIB)

The dual-beam instrument FEI-Helios NanoLab G3 UC (Thermo Fisher Scientific, Eindhoven, Netherlands) was used for the cross sections and direct imaging of the layers. The instrument combines a focused ion beam column (FIB, Ga+ ions, ion energy = 30 keV) with a high-resolution field emission scanning electron microscope (SEM) (Schottky Thermal Field Emitter). This device enables BSE imaging with high-contrast backscattered electrons using retractable detectors.

### Energy-dispersive X-ray spectrometry (EDX)

All energy dispersive X-ray analyses were done using a state of the art 30 mm^2^ silicon drift detector (SDD) by BRUKER (BRUKER Nano GmbH, Berlin, Germany) and the Esprit spectra evaluation software package. The specified energy resolution of the detector at 5.9 keV (Mn-Kα) is 129 eV.

### X-ray diffraction (XRD) and X ray reflectometry (XRR)

The XRD analysis has been performed with an X′pert Pro MPD Instrument theta-theta diffractometer (PANanalytical, Almelo, Netherlands) using Cu-Kα1,2 radiation. Used optics were a parallelmirror equipped with 1/8° divergence slit, 10 mm mask, 0.04 rad Sollerslit and 1.4 mm antiscatterslit. On the diffracted beam side, a 0.18° parallel plate collimator was used with a Panalytical PIXCEL1D detector in 0D mode with 255 channels. The samples were measured with gracing incidence XRD, fixed omega angle = 1.5°, 2theta range from 10° to 90° with 0.026° steps. Total measuring time was 7 h to 10 h. For phase identification software Malvern Panalytical HighScore Plus V.4.9 was used with databases ICDD, ICSD and COD. The Scherrer equation was used for the determination of the crystallite sizes. Supplementary Fig. 7 shows the XRD data of the electrochemically grown porous platinum.

The XRR thickness measurements were done with the diffractometer used also for XRD. For Ti-NiCr double-layer system the used optics was a parallelmirror (PM) (equipped with 1/8° divergence slit, 10 mm mask, 0.04 rad sollerslit, 1.4 mm antiscatterslit - Cu Ka1-radiation) or a Hybridmonochromator (HM) (equipped with 1/2° divergence slit and 2 mm mask – monochromatic Cu Ka1-radiation) on the incoming beam and a 0.18° parallel plate collimator with 0.1 mm receivingslit, PIXCEL1D detector in 0D mode with 5 or 3 channels for the highly reflecting Pt layer. The scan were omega-theta-scans from 0.04° to 5°(PM) or 0.04° to 3° (HM) omega with 0.05°-steps. Total scan time 1h40min (PM) or 30 min (HM). For thickness calculation software Malvern Panalytical AMASS V 1.1 was used.

### Ti layer thickness XRR evaluation

Ti layer thickness 8.0 nm (Fourier) – 9.0 nm (4 Frings)

The Fourier analysis of the reflectogram (Supplementary Figs. 1, 2) shows 3 peaks at 4.2 nm, 8 nm and 12.2 nm. Peak 1 (8 nm) comes from the Ti layer, peaks 2 and 3 are sum or difference peaks of an approximate 4 nm thick layer, presumably the SiO_2_ layer on the Si substrate.

### NiCr layer thickness XRR evaluation

NiCr layer thickness 102.3 nm (Fourier) – 102.9 nm (20 Frings)

The “small” frings were evaluated to determine the NiCr layer thickness (Supplementary Figs. 3, 4). In the reflectograms there are clearly visible frings in the 2nd and 3rd “large” frings (1/8° divergence slot, 1 h 40 min measuring time). Critical angle (deg): 0.3227 First fringe angle (deg): 0.7634 Second fringe angle (deg): 1.5826. Calculated Thickness (nm): 5.1469* 20 frings = 102.9 nm

### Highly reflecting Pt nanolayer thickness XRR evaluation

Reflectogram of the Pt nanolayer (Supplementary Fig. 5) enables an evaluation of the fringes thickness (nm): 6.3083 x 20 Frings = 126.2 nm. The Fourier plot (Supplementary Fig. 6) from the corresponding reflectogram indicates a Fourier Plot:Critical angle (deg): 0.5229, a Fourier start angle (deg): 0.0425 and a Fourier end angle (deg): 2.9975.

### Light microscopy

The micrographs of the electrodes were recorded with a Zeiss Axio light microscope using a 2.5NEOFLUAR objective.

### Ultraviolet (UV) irradiation

UV irradiation was performed using a Faust lamp (Sartorius lab Instruments, Switzerland) with an irradiation wavelength of 254 nm.

### Electrochemistry

Most of the electrochemical measurements were performed using a computer-assisted AUTOLAB Metrohm Potentiostat 302 N with FRA32M-module for impedance measurements equipped with customized electrodes. The CO_2_ reduction measurements were performed under ambient temperature and UV light exposure on cathode in an airtight electrochemical cell connected to a FTIR gas cell of 100 mL (Fig. 4G) equipped with KBr windows and mounted into a Bruker Vertex 80 V spectrometer. The cathode compartment consists of working electrode, while the anode compartment consists of platinum/Cu plate. The cathode was one by one: copper/copper oxide wire, porous platinum copper wire, porous platinum on rough conductive silicon, porous platinum on Pt plate. Each of the compartments loaded humid CO_2_ from ice-CO_2_. Between electrode compartments there is a humid Nafion proton exchange membrane, impermeable to oxygen. The electrochemical measurements were carried out under a difference of 2 V between electrodes under 0.1 A/cm^2^ while recording the spectra with a Bruker Vertex 80 V spectrometer.

### Electrocatalytic water electrolysis

**Scheme 1:**
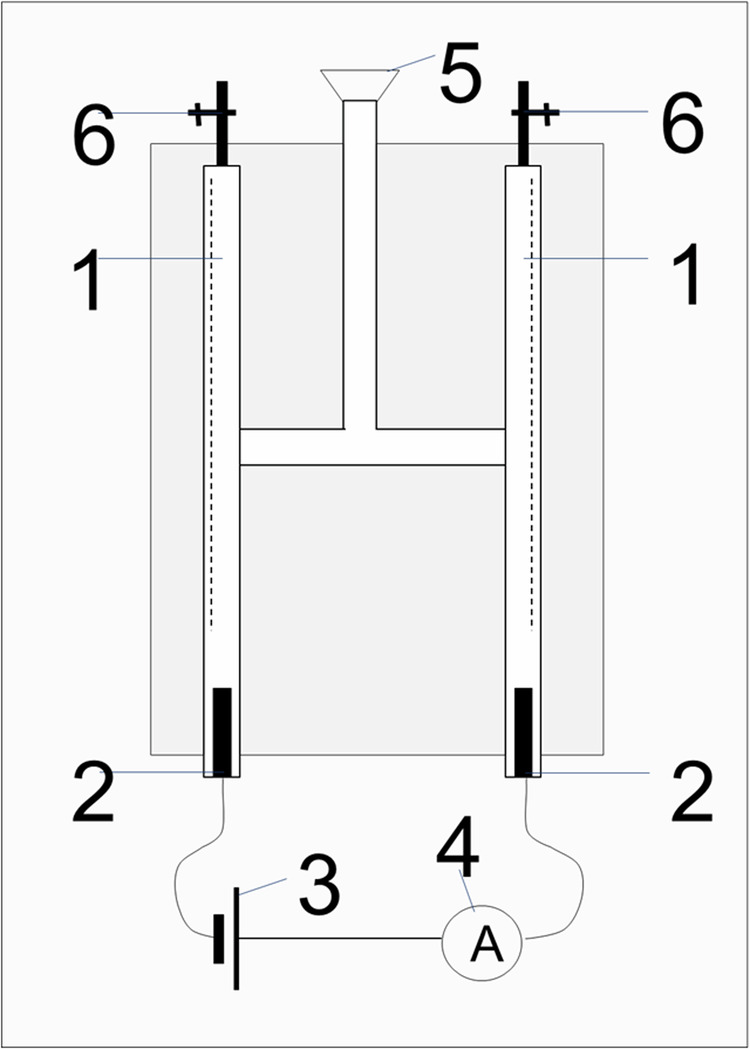
**Electrochemical setup**. Electrocatalytic production of oxygen and hydrogen from acidified water.1- gas burette; 2-porous platinum modified electrodes; 3-DC voltage source; 4-ampermeter; 5- funnel for acidified water; 6- taps.

With the setup from Scheme 1 the electrolysis of water is carried out. 0.1% H_2_SO_4_ is introduced into the electrolyzer and the circuit is closed. During the reaction, taps 6 must be closed to monitor the gases released in the two burettes. The reaction equations from the electrodes are:

13 $${2{{\rm{HO}}}}^{-}\,\to {0.5{{\rm{O}}}}_{2}+{{{\rm{H}}}}_{2}{{\rm{O}}}+{2{{\rm{e}}}}^{-}({{\rm{oxidation}}}\; {{\rm{at}}}\; {{\rm{the}}}\; {{\rm{anode}}}){{\rm{V}}}=+\,0.401{{\rm{V}}}$$

14 $${{{\rm{H}}}}^{+}\,+{{{\rm{e}}}}^{-}\to {0.5{{\rm{H}}}}_{2}({{\rm{reduction}}}\; {{\rm{at}}}\; {{\rm{the}}}\; {{\rm{cathode}}}){{\rm{V}}}=0.0{{\rm{V}}}\; {{\rm{vs}}}\; {{\rm{RHE}}}\; {{\rm{or}}}\; {{\rm{SHE}}}$$

The solution also holds sulfate ions SO_4_^2−^, which, due to the high potential required for discharge, remain not reduced. By measuring the intensity of the electric current through the circuit (approximately 0.3 A), respectively, the volumes of gases released in 10 min at the electrodes, one calculates the electrolysis yield:

15 $$\eta =\frac{{V}_{{real}}}{{V}_{{theoretical}}}{\blacktriangledown}100$$

Moreover, the calculation of faradaic efficiency uses the following relations: for CH_3_OH,

16 $${{FE}}_{C{H}_{3}{OH}}=\frac{{6{Fn}}_{C{H}_{3}{OH}}}{{It}}{\blacktriangledown}100 \%$$

for H_2_, where *F* is the Faraday constant, *n*_CH3OH_ is the moles of produced CH_3_OH, and *n*_H2_ for the produced H_2_:

17 $${{FE}}_{{H}_{2}}=\frac{{6{Fn}}_{{H}_{2}}}{{It}}{\blacktriangledown}100 \%$$

## Supplementary information

Supplementary Information

## Data Availability

The datasets generated and analyzed during the current study are available from the corresponding authors on reasonable request.
