# Supplementary material for: Electrochemically grown porous platinum for electrocatalysis and optical applications
Source: Commun Chem. 2025 Mar 29;8:93. doi: 10.1038/s42004-025-01476-4 (PMC11954909; doi:10.1038/s42004-025-01476-4)
Supplement: Supplementary file 1 — Supplementary Information [file 42004_2025_1476_MOESM1_ESM.pdf]

Supplementary Information

Electrochemically Grown Porous Platinum

for Electrocatalysis and Optical Applications

Sarmiza-Elena Stanca[1*] , Marco Diegel[1], Jan Dellith[1], Gabriel Zieger[1], Uwe Hübner[1], Andreas Ihring[1], Heidemarie Krüger[1,2]

[1]Leibniz Institute of Photonic Technology, Jena, Germany

[2]Institute for Solid State Physics, Friedrich-Schiller University Jena, Jena, Germany

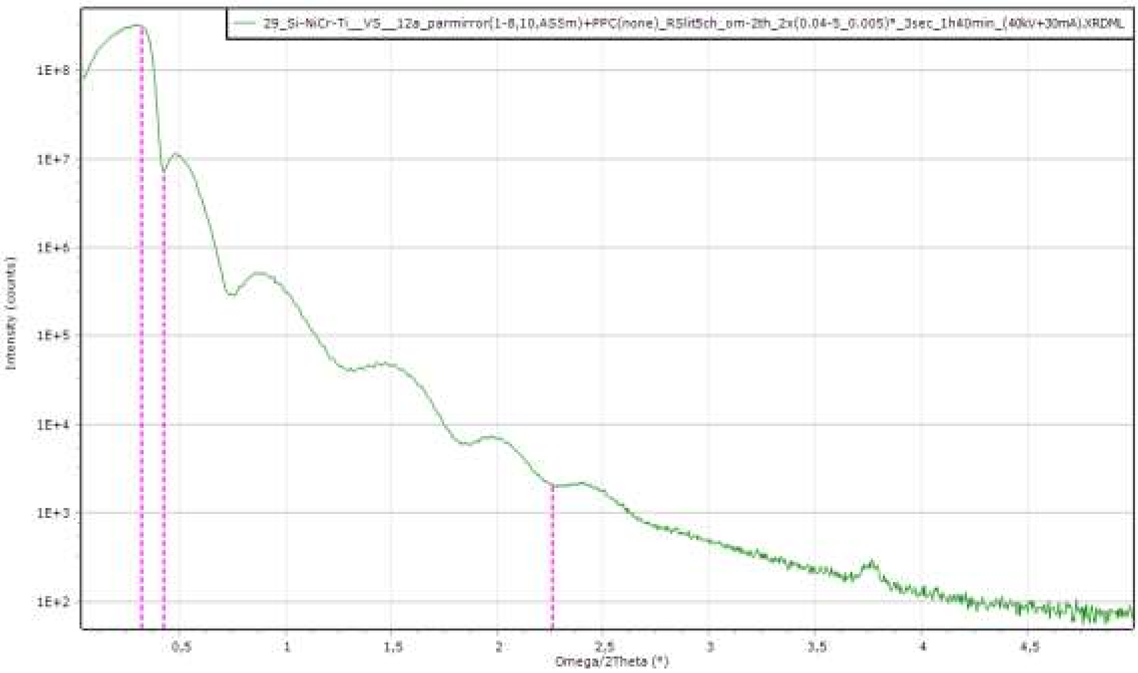

Supplementary Figure 1. XRR thickness evaluation of Ti layer. Reflectogram (1/8° divergence slot, 1h40min measuring time).Critical angle (deg): 0.3227 First fringe angle (deg): 0.428 Second fringe angle (deg): 2.2576. Calculated Thickness (nm): 2.2595 * 4 frings = 9.0 nm

*To whom correspondence should be addressed: sarmiza.stanca@leibniz-ipht.de.

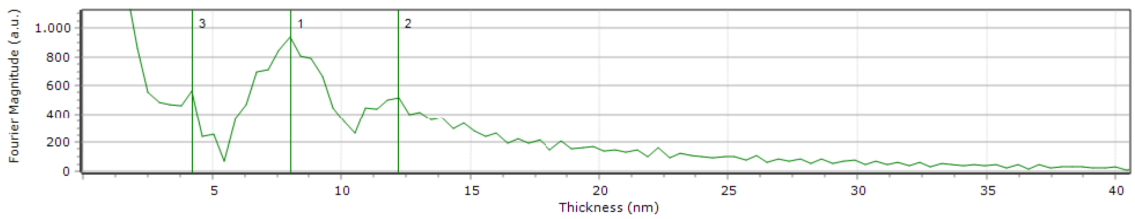

Supplementary Figure 2. Fourier plot of the reflectogram indicated in the Supplementary Figure 1. Critical angle (deg): 0.3227 Fourier start angle (deg): 0.043 Fourier end angle (deg): 4.997. Label: 3 Thickness (nm): 4.2; Label: 1 Thickness (nm): 8; Label: 2 Thickness (nm): 12.2. NiCr layer thickness Frings evaluation**.**The "small" frings were evaluated to determine the NiCr layer thickness. In the reflectogram there are clearly visible frings in the 2nd and 3rd "large" frings.

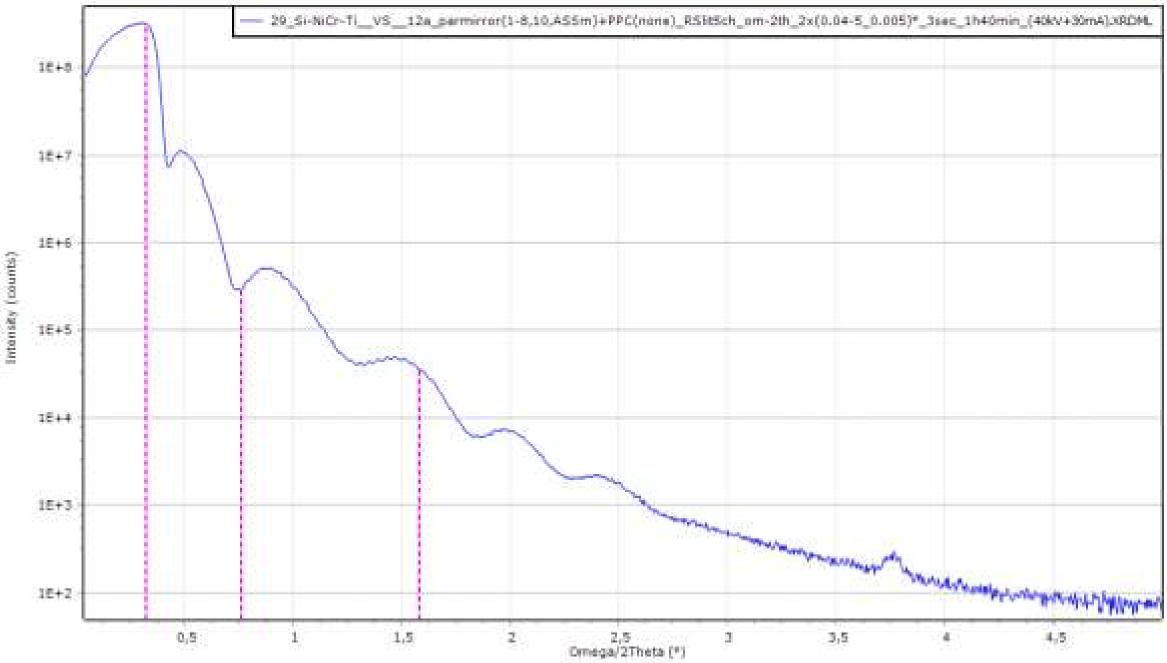

Supplementary Figure3. XRR thickness evaluation of NiCr layer. Reflectogram (1/8° divergence slot, 1h40min measuring time).Critical angle (deg): 0.3227 First fringe angle (deg): 0.7634 Second fringe angle (deg): 1.5826. Calculated Thickness (nm): 5.1469* 20 frings = 102.9 nm

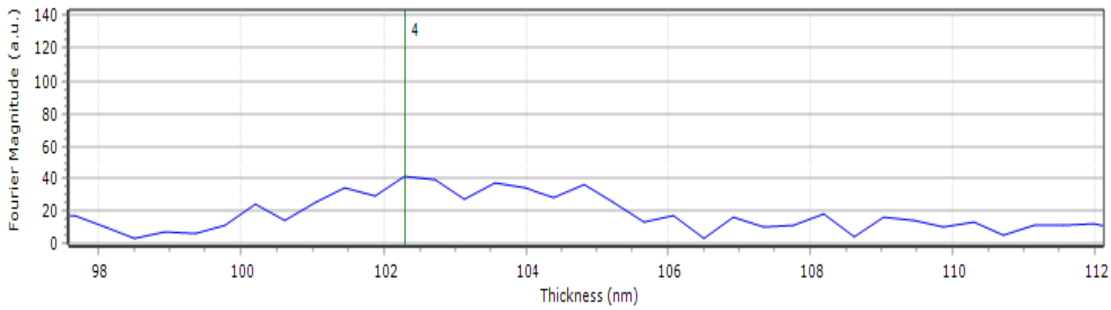

Supplementary Figure 4. Fourier plot of the reflectogram indicated in the Supplementary Figure 3.

Critical angle (deg): 0.3227 Fourier start angle (deg): 0.043 Fourier end angle (deg): 4.997. Label: 4

Thickness (nm): 102.3

1

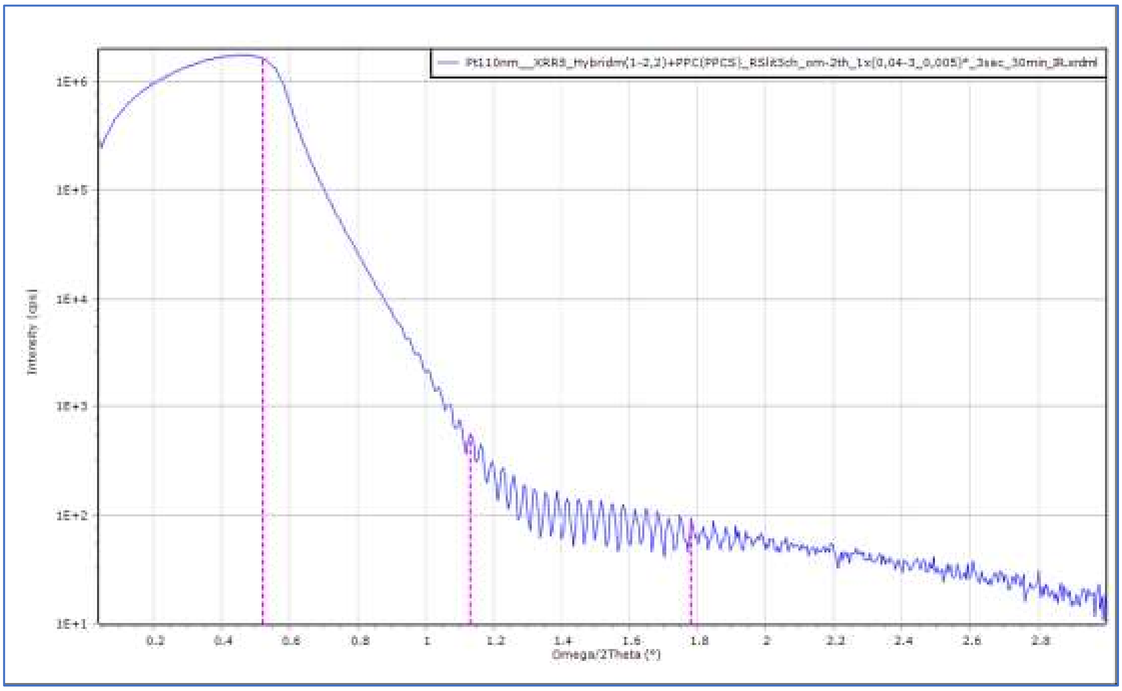

Supplementary Figure 5: Reflectogram of the Pt nanolayer on silicon substrate.

Critical angle (deg): 0.5229 First fringe angle (deg): 1.1324 Second fringe angle (deg): 1.7825

Calculated FRINGS Thickness (nm): 6.3083 x 20 Frings = 126.2 nm.

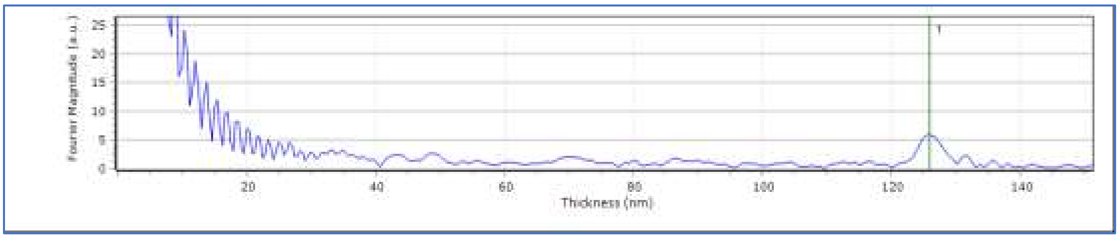

Supplementary Figure 6. Fourier plot from the reflectogram indicated in the Supplementary Figure 5. Thickness Analysis Fourier Plot:Critical angle (deg): 0.5229 Fourier start angle (deg): 0.0425 Fourier end angle (deg): 2.9975. Thickness (nm): 125.9.

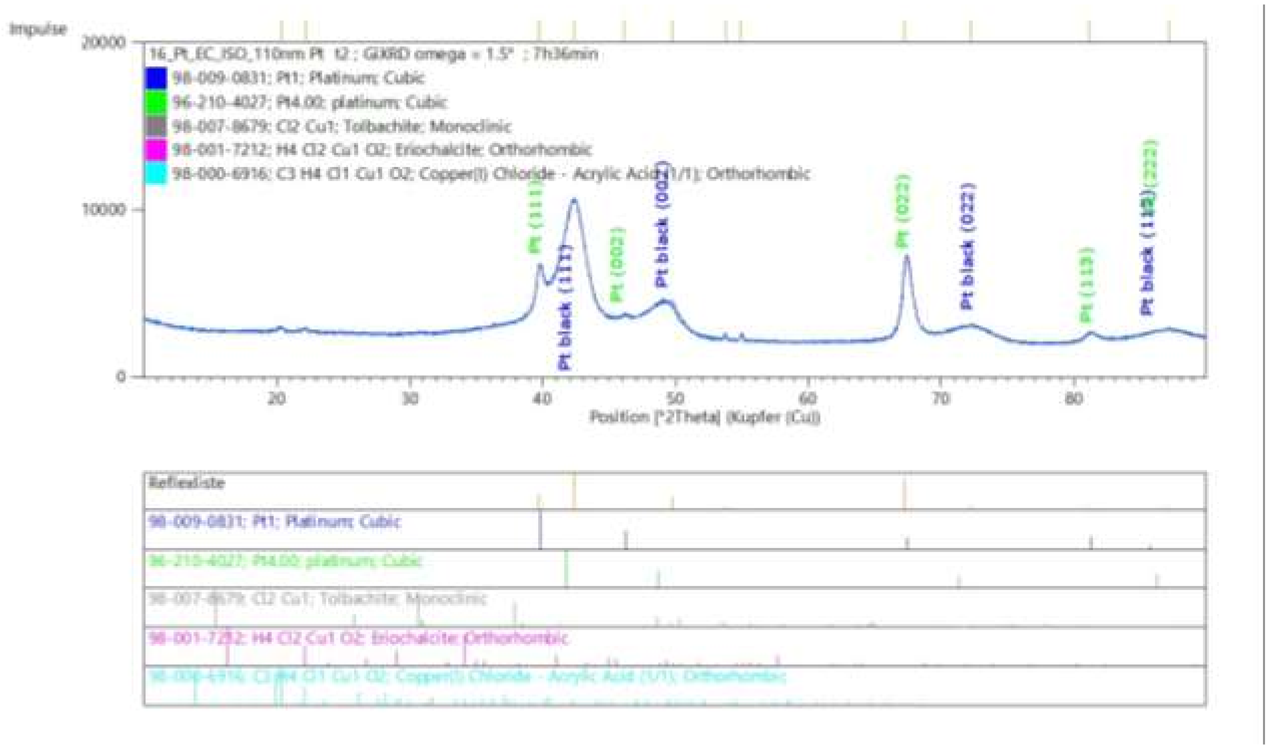

Supplementary Figure 7. X-Ray diffraction data of the electrochemically grown porous platinum. Thickness (nm): 125.9.
